# Sensitization of colonic nociceptors by IL-13 is dependent on JAK and p38 MAPK activity

**DOI:** 10.1152/ajpgi.00280.2022

**Published:** 2023-02-07

**Authors:** Katie H. Barker, James P. Higham, Luke A. Pattison, Iain P. Chessell, Fraser Welsh, Ewan St. J. Smith, David C. Bulmer

**Affiliations:** ^1^Department of Pharmacology, University of Cambridge, Cambridge, United Kingdom; ^2^Department of Neuroscience, BioPharmaceuticals R&D, AstraZeneca, Cambridge, United Kingdom

**Keywords:** IL-13, inflammatory bowel disease, JAK, nociception, p38 MAPK

## Abstract

The effective management of visceral pain is a significant unmet clinical need for those affected by gastrointestinal diseases, such as inflammatory bowel disease (IBD). The rational design of novel analgesics requires a greater understanding of the mediators and mechanisms underpinning visceral pain. Interleukin-13 (IL-13) production by immune cells residing in the gut is elevated in IBD, and IL-13 appears to be important in the development of experimental colitis. Furthermore, receptors for IL-13 are expressed by neurons innervating the colon, though it is not known whether IL-13 plays any role in visceral nociception per se. To resolve this, we used Ca^2+^ imaging of cultured sensory neurons and ex vivo electrophysiological recording from the lumbar splanchnic nerve innervating the distal colon. Ca^2+^ imaging revealed the stimulation of small-diameter, capsaicin-sensitive sensory neurons by IL-13, indicating that IL-13 likely stimulates nociceptors. IL-13-evoked Ca^2+^ signals were attenuated by inhibition of Janus (JAK) and p38 kinases. In the lumbar splanchnic nerve, IL-13 did not elevate baseline firing, nor sensitize the response to capsaicin application, but did enhance the response to distention of the colon. In line with Ca^2+^ imaging experiments, IL-13-mediated sensitization of the afferent response to colon distention was blocked by inhibition of either JAK or p38 kinase signaling. Together, these data highlight a potential role for IL-13 in visceral nociception and implicate JAK and p38 kinases in pronociceptive signaling downstream of IL-13.

## Graphical abstract

**Figure:**
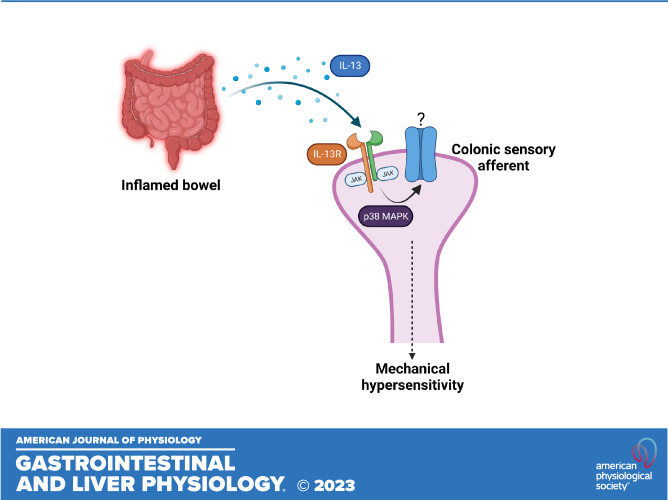

## INTRODUCTION

Proinflammatory cytokines can directly stimulate and/or sensitize dorsal root ganglion (DRG) neurons, as well as sensitizing colonic afferent responses to noxious stimuli, thereby implicating these mediators in hypersensitivity and pain in gastrointestinal (GI) disease. Although much research has already been conducted to determine the role of cytokines like tumor necrosis factor α (TNFα) in pain signaling pathways, some mediators remain poorly understood. One such cytokine is interleukin-13 (IL-13), a member of the T helper (Th) 2 family of cytokines, alongside IL-3, IL-4, IL-5, and IL-9. In humans, IL-13 is a 10–14-kDa immunoregulatory protein produced by activated CD4^+^ T cells and immune cells like mast cells, eosinophils, and basophils (1–3). The gene encoding IL-13 in humans is located on chromosome 5q31 and consists of four exons and three introns, just upstream of the gene encoding IL-4 (4, 5). Although IL-13 and IL-4 only share 25% similarity at the amino acid level, the two cytokines share overlapping roles in allergic inflammation and the response to parasite infection (6–8).

Given the overlapping physiological roles of IL-4 and IL-13, it should perhaps be no surprise that they also share similar signaling pathways, with both cytokines activating the heterodimeric type II receptor, which consists of a 140 kDa IL-4Rα and a 65–70 kDa IL-13Rα1 subunit (9, 10). IL-13Rα1 monomers bind IL-13 with low affinity, but dimerization with IL-4Rα increases binding affinity and receptor functionality (11, 12). Although the IL-4Rα subunit is also found together with the γc subunit in the type I receptor that interacts exclusively with IL-4 (13), IL-13 similarly binds with higher affinity to the IL-13Rα2 receptor, which is often referred to as a decoy receptor owing to a lack of signal transduction following IL-13 binding (10).

The different receptor subunits of IL-4 and IL-13 receptors (other than IL-13Rα) activate common downstream signaling pathways on cytokine binding. For example, the IL-4Rα receptor complexes are closely associated with Janus kinase (JAK) proteins; JAK1 associates with the IL-4Rα chain, JAK2/tyrosine kinase 2 (Tyk2) with IL-13Rα1, and JAK3 with γc (14–16). JAK proteins phosphorylate IL-4Rα and recruit signal transducer and activator of transcription 6 (STAT6; 17, 18). Once phosphorylated, STAT6 translocates to the nucleus (14–16), but this interaction may also lead to activation of phosphatidylinositol 3-kinase (PI3K) and MAPK signaling pathways, as demonstrated in a range of in vitro systems (19–22).

IL-13 production is elevated in gastrointestinal (GI) diseases, with increased production by natural killer T (NKT) cells (23, 24). Moreover, other cell types within the gut, for example, innate lymphoid cells (ILCs) and mast cells, can be stimulated to produce IL-13 by gut epithelium-derived cytokines, such as IL-33 and IL-25, which themselves are produced during inflammation and infection (25–28). Increased secretion of IL-13 via this mechanism may in part induce and maintain chronic idiopathic inflammation within the GI tract.

In experimental mouse models of colitis, IL-4 and IL-13 production contributes to disease pathogenesis with levels increasing progressively from pre- to early- to late-stage disease (29). Moreover, neutralization of IL-13 by IL-13Rα2-Fc administration (30) or use of a bifunctional therapeutic targeting IL-4 and IL-13 (31) prevented oxazolone-induced colitis in mouse models. In dextran sodium sulfate (DSS)-induced colitis, IL-13 secretion is also upregulated (32) and disease severity has also been linked to mucosal levels of IL-13 (33).

In inflammatory bowel disease (IBD) patients, however, the role of IL-13 is less clear, with contrasting results reported from patient samples. Early studies reported reduced IL-13 concentrations in the inflamed mucosa of patients with ulcerative colitis (UC) compared with active Crohn’s disease (CD) and healthy controls (34), and more recently this has been reinforced by data showing similar levels of mucosal IL-13 mRNA and IL-13 protein production in active UC, inactive UC, and healthy controls (35). However, ex vivo stimulation of lamina propria T cells taken from resected specimens of patients with UC produced increased protein levels of IL-13 compared with stimulation of cells isolated from CD and healthy individuals (23, 36). In support of this, increased levels of IL-13Rα1 are present in ex vivo intestinal epithelial cells isolated from patients with UC (37) and increased expression of phosphorylated STAT6 has been identified in active UC with respect to CD and healthy controls (38). Although other signaling pathways may activate STAT6 (39), IL-4 and IL-13 are by far the primary drivers of STAT6 phosphorylation (14).

Thus far, IL-13 has been identified as a key driver in allergic asthma and has therefore influenced the production of therapeutics targeting the IL-13 pathway, including antibodies like anrukinzumab, lebrikizumab, and tralokinumab, which prevent IL-13 signaling through IL-13Rα1 and IL-13Rα2, while maintaining IL-4 signaling through the type I receptor (40). Although these strategies have been clinically successful in patients with moderate-to-severe asthma (41), clinical trials in patients with IBD have been inconclusive. IL-13 neutralization by the human IgG4 monoclonal antibody tralokinumab increases the rate of clinical remission in patients with UC compared with controls (42), whereas anrukinzumab, a humanized IgG1 antibody, failed to alter clinical symptoms or induce remission (43).

IL-13 has recently been shown to directly activate sensory neurons, which express IL-13Rα1 and IL-4Rα, and activation of neuronal IL-4Rα by IL-13 sensitizes sensory neurons to multiple other compounds (44). In colonic sensory neurons, transcript levels for both IL-13Rα1 and IL-4Rα are abundant and levels correlate with TRPV1 transcript levels, suggesting that IL-13 has the ability to interact with nociceptors innervating the colon (45). Furthermore, transient receptor potential (TRP) channel-dependent IL-13 signaling has been linked to p38 MAPK and JAK activation (44), with increasing use of JAK inhibitors in the clinic to reduce visceral pain in GI disease (46). We, therefore, hypothesized that the primary agonist of the type II IL-4R, IL-13, sensitizes colonic nociceptors to noxious mechanical distension and capsaicin application, the mechanism for which is dependent on p38 MAPK and JAK signaling pathways.

## MATERIALS AND METHODS

### Ethical Approval

All animal experiments were conducted in compliance with the Animals (Scientific Procedures) Act 1986 Amendment Regulations 2012 under Project License P7EBFC1B1 granted to E. St. J. Smith by the Home Office with approval from the University of Cambridge Animal Welfare Ethical Review Body.

### Reagents

Stock concentrations of IL-13 [1 µM; H_2_O with 0.1% (wt/vol) bovine serum albumin] and capsaicin (1 mM; 100% ethanol) were purchased from ThermoFisher and Sigma-Aldrich, respectively and dissolved as described. SB203580 (10 mM; DMSO; Tocris, Cat. No. 1402), pyridone 6 (1 mM; DMSO; Tocris, Cat. No. 6577), and ruxolitinib (1 mM; DMSO; Tocris, Cat. No. 7064) were purchased from Tocris and stock concentrations made up as described. Nifedipine (100 mM; DMSO) and atropine (100 mM; 100% ethanol) were purchased from Sigma-Aldrich and dissolved as described. All drugs were diluted to working concentrations in extracellular solution (ECS) or Krebs buffer on the day of the experiment.

### Animals

Adult male C57BL/6J mice (8–16 wk) were obtained from Charles River (Cambs, UK; RRID:IMSR_JAX:000664). Mice were conventionally housed in temperature-controlled rooms (21°C) with a 12-h light/dark cycle and provided with nesting material, a red plastic shelter and access to food and water ad libitum.

### Primary Culture of Mouse Dorsal Root Ganglion Neurons

DRG neurons were cultured as previously described (47). In brief, mice were humanely euthanized by exposure to a rising concentration of CO_2_, with confirmation by cervical dislocation. The spine was removed and bifurcated to allow isolation of DRG neurons. Spinal DRG neurons innervating the distal colon (T12-L5) were removed into ice-cold Leibovitz’s L-15 Medium, GlutaMAX Supplement [supplemented with 2.6% (vol/vol) NaHCO_3_]. DRG neurons were then incubated with 1 mg/mL collagenase (15 min) followed by 30 min trypsin (1 mg/mL) both with 6 mg/mL bovine serum albumin (BSA) in Leibovitz’s L-15 Medium, GlutaMAX Supplement [supplemented with 2.6% (vol/vol) NaHCO_3_]. After the enzymes were removed, DRG neurons were resuspended in 2-mL Leibovitz’s L-15 Medium, GlutaMAX Supplement containing 10% (vol/vol) fetal bovine serum (FBS), 2.6% (vol/vol) NaHCO_3_, 1.5% (vol/vol) glucose, and 300 U/mL penicillin and 0.3 mg/mL streptomycin (P/S). DRG neurons were mechanically triturated using a 1-mL Gilson pipette and centrifuged (1,000 rpm) for 30 s. The supernatant, containing dissociated DRG neurons, was collected in a separate tube and the pellet resuspended in 2-mL Leibovitz’s L-15 Medium, GlutaMAX Supplement containing 10% (vol/vol) FBS, 2.6% (vol/vol) NaHCO_3_, 1.5% (vol/vol) glucose, and P/S. This process of mechanical trituration was repeated six times, after which the total supernatant was centrifuged (1,000 rpm) for 5 min to pellet the dissociated DRG neurons. Cells were resuspended in 250-µL Leibovitz’s L-15 Medium, GlutaMAX Supplement containing 10% (vol/vol) FBS, 2.6% (vol/vol) NaHCO_3_, 1.5% (vol/vol) glucose, and P/S and plated (50 µL/dish) onto 35 mm glass bottomed dishes coated with poly-d-lysine (MatTek, Ashland, MA) and laminin (Thermo Fisher: 23017015). Dishes were incubated for 3 h (37°C, 5% CO_2_) to allow cell adhesion and subsequently flooded with Leibovitz’s L-15 Medium, GlutaMAX Supplement containing 10% (vol/vol) FBS, 2.6% (vol/vol) NaHCO_3_, 1.5% (vol/vol) glucose, and P/S and incubated overnight.

### Ca^2+^ Imaging

Extracellular solution (ECS; in mM: 140 NaCl, 4 KCl, 1 MgCl_2_, 2 CaCl_2_, 4 glucose, 10 HEPES) was prepared and adjusted to pH 7.4 using NaOH and an osmolality of 290–310 mosmol/kg H_2_O with sucrose. Medium was aspirated from DRG neuronal cultures, and cells were incubated (30 min) with 100 µL of 10 μM Ca^2+^ indicator Fluo-4-AM diluted in ECS (room temperature; shielded from light). For inhibitor studies requiring preincubation, 200 µL of drug was added for 10 min before imaging.

Dishes were mounted on the stage of an inverted microscope (Nikon Eclipse TE-2000S), and cells were visualized with bright-field illumination at ×10 magnification. To ensure a rapid exchange of solutions during protocols, the tip of a flexible perfusion onflow tube (AutoMate Scientific, CA) attached to a six-channel, gravity-fed perfusion system (Warner Instruments, CT) was placed beside the field of view. Initially, cells were superfused with ECS or drug in inhibitor studies to establish baseline.

Fluorescent images were captured with a charge-coupled device (CCD) camera (Rolera Thunder, Qimaging, MC, Canada or Retiga Electro, Photometrics, AZ) at 2.5 fps with 100 ms exposure. Fluo-4 was excited by a 470 nm light source (Cairn Research, Faversham, UK). Emission at 520 nm was recorded with μManager (48). All protocols began with a 10 s baseline of ECS before mediator superfusion, after which neurons were washed with ECS for at least 20 s to remove the mediator. With multiple drug additions to the same dish, cells were allowed 4 min recovery between applications. Finally, cells were stimulated with 50 mM KCl for 10 s to determine cell viability, identify neuronal cells and allow normalization of fluorescence. A fresh dish was used for each protocol, and all solutions were diluted in ECS.

### Ca^2+^ Imaging Data Analysis

Regions of interest were circled from a bright-field image and outlines overlaid onto fluorescent images using ImageJ (NIH, Bethesda, MD). Pixel intensity was measured and analyzed with custom-written scripts in RStudio (RStudio, MA) to compute the proportion of neurons stimulated by each drug application and the corresponding magnitude of response. Briefly, the background fluorescence was subtracted from each region of interest, and fluorescence intensity (*F*) baseline corrected and normalized to the maximum fluorescence elicited during 50 mM KCl stimulation (*F*_pos_). Maximum KCl fluorescence was denoted as 1 *F*/*F*_pos_. Further analysis was confined to cells with a fluorescence increase ≥5 standard deviations above the mean baseline after 50 mM KCl application, as these were considered viable neurons. Using manual quality control, neurons were deemed responsive to drug application if a fluorescence increase of 0.1 *F*/*F*_pos_ was observed in response to drug perfusion. Responses to drug application were discounted if the increase in fluorescence began before or after the period of drug perfusion. The proportion of responsive neurons and magnitude of the fluorescence response was measured for each experiment, with peak responses calculated from averaging fluorescence values of individual neurons at each time point. Cell diameter was also measured with ImageJ software to allow size comparison of responsive neurons.

### Ex Vivo Whole Nerve Electrophysiological Recordings of Colonic Splanchnic Afferents

Lumbar splanchnic nerve (LSN) afferent preparations were conducted as previously described (47). Briefly, the distal colorectum (splenic flexure to rectum) and associated LSN (rostral to inferior mesenteric ganglia) were isolated from mice euthanized as described earlier. The colon was cannulated with fine thread (polyester, Gutermann) in a rectangular recording chamber with Sylgard base (Dow Corning, UK) and serosally superfused (7 mL/min; 32°C–34°C) and luminally perfused (200 μL/min) by a syringe pump (Harvard apparatus, MA) with carbogenated Krebs buffer solution (95% O_2_–5% CO_2_). Krebs buffer was supplemented with 10 μM atropine and 10 μM nifedipine to prevent smooth muscle activity (49).

Borosilicate suction electrodes were used to record the multiunit activity of LSN bundles. Signals were amplified (gain 5 kHz), band pass filtered (100–1,300 Hz; Neurolog, Digitimer Ltd, UK), and digitally filtered for 50 Hz noise (Humbug, Quest Scientific, Canada). Analog signals were digitized at 20 kHz (Micro1401; Cambridge Electronic Design, UK), and signals were visualized with Spike2 software (Cambridge Electronic Design, UK).

### Electrophysiology Protocols

After dissection, preparations underwent a minimum 30 min stabilization period to ensure baseline firing was consistent. Repeated ramp distensions (0–80 mmHg) were performed on the cannulated distal colon by occluding the luminal perfusion outflow and increasing intraluminal pressure gradually over ∼220 s. When the maximum 80 mmHg pressure was achieved, the luminal outflow was reopened and pressure returned to baseline. Distension pressures >30 mmHg evoke noxious pain behaviors in mice and humans (50, 51).

Each protocol consisted of five ramp distensions, separated by 15 min to allow baseline stabilization, after which the colon was superfused with 1 µM capsaicin (20 mL). Between ramps three and four, mediator (100 nM IL-13) or vehicle (buffer) was applied by luminal perfusion for 15 min. In protocols examining the effects of pathway inhibition, preparations were pretreated with inhibitor [10 µM SB203580 (52) or 1 µM Pyridone 6 (53)] or vehicle (0.01% DMSO) for 15 min before and throughout mediator perfusion.

### Electrophysiological Data Analysis

Nerve discharge was determined by quantifying the number of spikes passing through a manually determined threshold, twice the level of background noise (typically 60–80 μV) and binned to determine the average firing frequency over 10 s. Baseline firing was established by the average activity 180 s before distension or drug perfusion. Changes to neuronal firing rates were calculated by subtracting baseline activity from increases in nerve activity in response to ramp distension or perfusion with capsaicin. Peak firing to noxious mechanical distension and capsaicin application was defined respectively as the highest neuronal activity during the fifth ramp distension and during the 10 min postcapsaicin application. Changes to neuronal activity during ramp distension were measured at each 5 mmHg increase in pressure and used to visualize ramp profiles. Response profiles to capsaicin application were plotted from binned data averaged from 30-s increments. The area under the curve (AUC) was calculated for the duration of each ramp distension (0–80 mmHg) and for the 10-min post initial capsaicin application following the generation of response profiles using GraphPad Prism 9 software (GraphPad Software).

### Statistical Analysis

All data sets were tested for normality with Shapiro–Wilk’s test, and, based on the results, the appropriate parametric or nonparametric analysis was used. All data are displayed as means ± standard deviation (SD). For Ca^2+^ imaging analysis, *n* represents the number of dishes imaged and *N* represents the total number of mice from which they were cultured. On average, ∼180 neurons were imaged per dish. For analysis of ex vivo electrophysiology, *N* represents the number of mice from which nerve recordings were made. One recording was made per animal. *P* values are represented in figures as *,&,#*P* < 0.05, **,&&,##*P* < 0.01, ***,&&&*P* < 0.001.

## RESULTS

### IL-13 Increases [Ca^2+^]_i_ in DRG Sensory Neurons Cosensitive to Capsaicin

**Figure 1.**
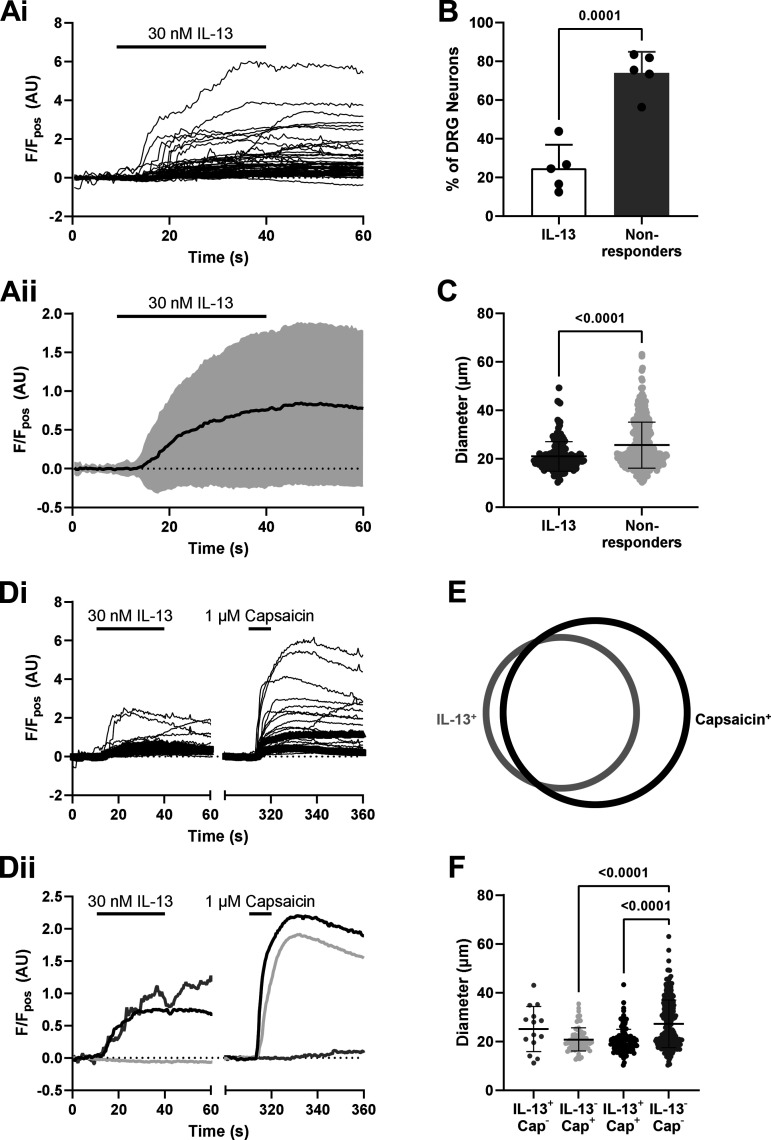
IL-13 increases [Ca^2+^]_i_ in capsaicin-sensitive DRG neurons. *A*: representative traces from individual (*i*) and averaged (*ii*) neuronal responses to 30 nM IL-13 (*n* = 63 neurons). *B*: the percentage of DRG neurons stimulated by IL-13 was significantly lower than nonresponsive neurons. *C*: IL-13-sensitive neurons were smaller in diameter than nonresponders (*n* = 644 neurons). *D*: example traces from (*i*) individual neurons responsive to both IL-13 and 1 µM capsaicin and (*ii*) response profiles of responder populations (black, IL-13^+^/cap^+^; dark gray, IL-13^+^/cap^−^, light gray: IL-13^−^/cap^+^). *E*: representative Venn diagram of overlapping responses in neurons stimulated with IL-13 and capsaicin (*n* = 5, *N* = 5). *F*: size distribution of IL-13- and/or capsaicin-sensitive neurons. IL/13/capsaicin cosensitive and capsaicin-sensitive neurons were smaller in diameter than nonresponders (*n* = 488 neurons). DRG, dorsal root ganglion.

Previous studies investigating the role of IL-13 in itch sensory pathways have shown its ability to directly activate DRG sensory neurons (44). To corroborate these findings and the extent to which IL-13 stimulated DRG sensory neurons, 30 nM IL-13 was applied to neurons and the change in [Ca^2+^]_i_ measured using Fluo4. As expected, IL-13 increased [Ca^2+^]_i_ as demonstrated by an increase in Fluo4 fluorescence (Fig. 1*A*). Of the total neurons assessed, IL-13 activated 24.8 ± 12.1%, significantly fewer than those that were IL-13-insensitive (*P* = 0.0001, unpaired *t* test; *n* = 5, *N* = 5; Fig. 1*B*). Comparison of cell size also revealed that IL-13 stimulated a population of smaller diameter neurons than those that were nonresponsive (*P* < 0.0001, Kolmogorov–Smirnov test; *n* = 644 neurons; Fig. 1*C*), suggesting that IL-13 was preferentially stimulating nociceptors. To investigate this further, IL-13-sensitive neurons were evaluated for their cosensitivity to 1 µM capsaicin (Fig. 1*Di*). Three populations of responsive neurons were identified, including those that were cosensitive to capsaicin and IL-13 (Fig. 1*Dii*). In total, 27.3 ± 11.2% of neurons were activated by IL-13, 89.1% of which were also sensitive to capsaicin (Fig. 1*E*). Overall, 40.8 ± 9.26% of neurons were sensitive to capsaicin, but only 59.7% of these were activated by IL-13, suggesting that IL-13 does not stimulate all nociceptors. This is corroborated by cell diameter data showing that neurons responsive to capsaicin alone (*P* < 0.0001) and those with overlapping sensitivity to capsaicin and IL-13 were smaller than neurons unresponsive to both IL-13 and capsaicin (*P* < 0.0001, Kruskal–Wallis test with Dunn’s multiple comparison; *n* = 488 neurons; Fig. 1*F*).

### IL-13-Mediated Ca^2+^ Signals in Sensory Neurons Are Dependent on JAK and p38 MAPK Signaling

**Figure 2.**
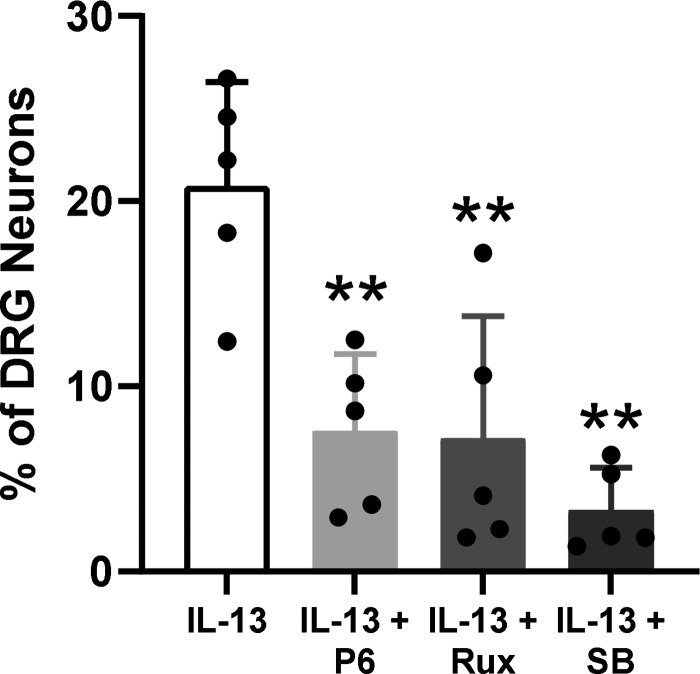
Activation of DRG sensory neurons by IL-13 is JAK1/2- and p38 MAPK-dependent. The proportion of IL-13-sensitive neurons was significantly reduced by pan-JAK inhibition with pyridone 6 (P6) (*n* = 5), JAK1/2 inhibition with ruxolitinib (Rux) (*n* = 5), and p38 MAPK inhibition with SB203580 (SB) (*n* = 5). DRG, dorsal root ganglion. **P < 0.01.

Activation of IL-13 receptor components IL-4Rα or IL-13Rα initiates transphosphorylation of JAK proteins and initiates signal transduction in a range of cell types, including immune cells (16, 54–57). In sensory neurons, activation of IL-4Rα by IL-4 is dependent on JAK1 signaling (44). To determine whether JAK signaling is also involved in the activation of DRG sensory neurons by IL-13, cultures were incubated with the pan-JAK inhibitor, pyridone 6 (1 µM). JAK inhibition decreased the proportion of IL-13-responsive neurons from 20.8 ± 5.62% to 7.58 ± 4.17% (*P* = 0.003, one-way ANOVA with Holm–Šídák’s multiple comparisons test; *n* = 5; Fig. 2*A*). Afterward, neurons were incubated with 1 µM ruxolitinib, a selective JAK1/2 inhibitor, to help establish more specifically which members of the JAK family were involved in IL-13-mediated increases in [Ca^2+^]_i_. Ruxolitinib reduced the proportion of IL-13 responders, with 7.20 ± 6.60% of neurons still activated (*P* = 0.002, one-way ANOVA with Holm–Šídák’s multiple comparisons test; *n* = 5; Fig. 2*A*), which was equivalent to pyridone 6 treatment (*P* > 0.999, one-way ANOVA with Holm–Šídák’s multiple comparisons test; *n* = 5), suggesting that JAK1 and JAK2 are involved in DRG sensory neuron activation by IL-13.

Aside from JAK signaling, p38 MAPK phosphorylation/activation has also been observed in immune cells following exposure to IL-13 and, in mast cells, IL-13 production is p38 MAPK-dependent, forming a feedback loop during inflammation (58, 59). To investigate if there was a role for p38 MAPK in IL-13 signaling in DRG sensory neurons, the p38 MAPK inhibitor SB203580 (10 µM) was added to culture dishes. In line with those observations in immune cells, p38 MAPK inhibition reduced the proportion of IL-13 responsive neurons by 85% (*P* = 0.0002, one-way ANOVA with Holm–Šídák’s multiple comparisons test; *n* = 5; Fig. 2*A*).

### IL-13 Sensitizes Colonic Afferent Responses to Noxious Distension via JAK and p38 MAPK

**Figure 3.**
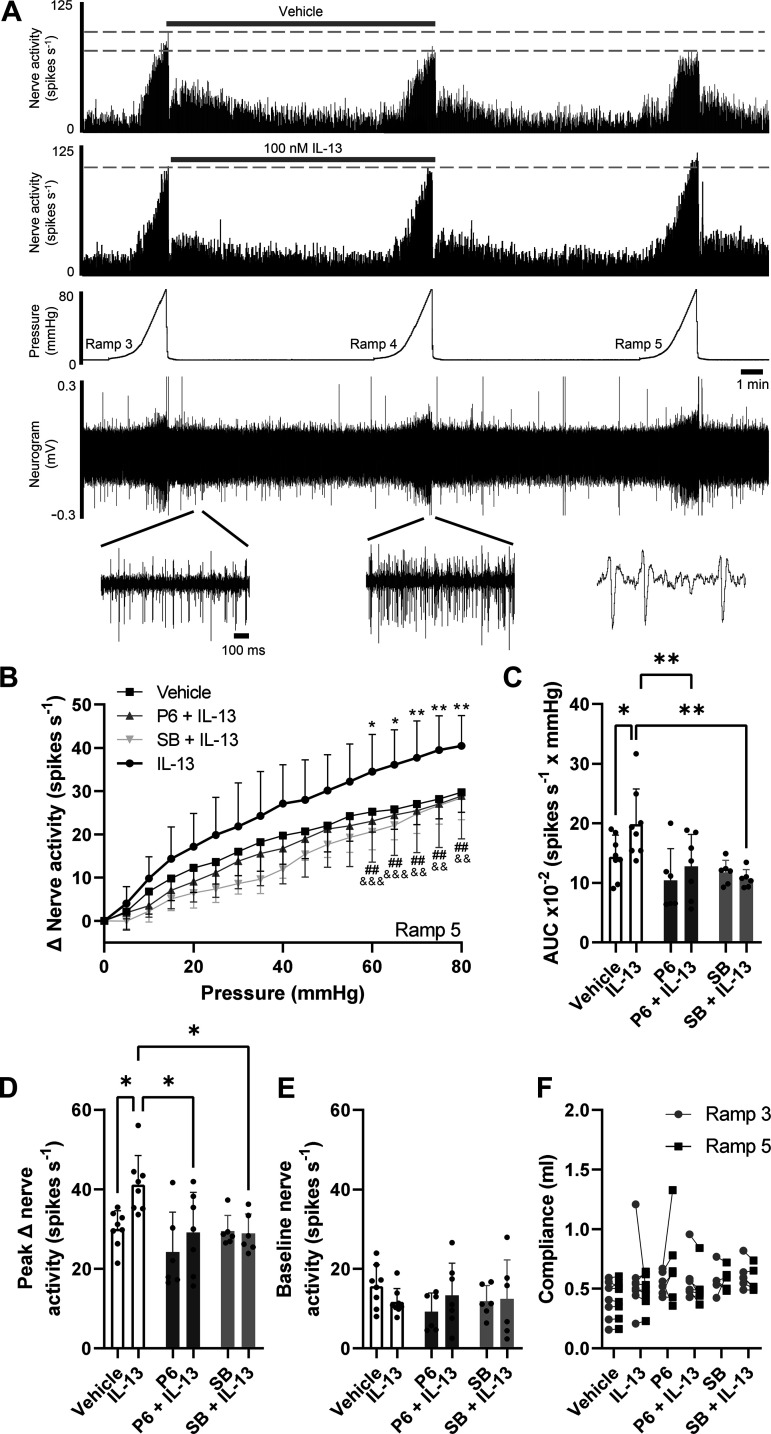
IL-13 evokes colonic afferent hypersensitivity to ramp distension via JAK and p37 MAPK. *A*: example rate histograms and neurograms showing LSN activity with accompanying pressure trace showing sequential (x3) ramp distensions (0–80 mmHg) from vehicle- and IL-13 (100 nM)-treated preparations, demonstrating the sensitization of colonic afferent responses to noxious distension after IL-13 perfusion. *B*: IL-13 perfusion shifted the afferent pressure-response curve (*Ramp 5*) at distension pressures greater than 60 mmHg compared with vehicle (*P < 0.05, **P < 0.01, vehicle vs. IL-13), whereas coapplication of either P6 (##*P* < 0.01, IL-13 vs. IL-13 + P6) or SB (&&*P* < 0.01, &&&*P* < 0.001 IL-13 vs. IL-13 + SB) abrogated the effect of IL-13. Application of P6 or SB alone had no effect on the pressure-response curve compared with vehicle (curves not shown for clarity, but these groups were included in two-way analysis). *C*: IL-13 potentiated the area under the pressure-response curve (AUC) compared with vehicle. This effect of IL-13 on distension-evoked afferent firing was abolished by incubation of the tissue with P6 or SB. Comparisons to the IL-13-treated group: *P < 0.05, **P < 0.01. *D*: peak afferent activity was significantly increased following IL-13 pretreatment; an effect abolished by coapplication of P6 or SB. Comparisons to the IL-13-treated group: *P < 0.05. *E*: spontaneous baseline afferent activity was indistinguishable between all treatment groups. *F*: colonic compliance was unchanged between sequential ramp distensions within treatment groups and no differences between treatment groups were observed. LSN, lumbar splanchnic nerve.

Stable afferent responses of the lumbar splanchnic nerve (LSN) during colonic distension (steadily ramped from 0 to 80 mmHg) were achieved after 2–3 distensions (Fig. 3*A*). Thus *Ramp 3* was used as a control ramp in each protocol, after which tissues were perfused with 100 nM IL-13 for 15 min and the effects on the LSN response to mechanical distension observed. In vehicle-treated tissues, no significant difference was observed in the area under the pressure-response curve (AUC; an indicator of total afferent activity during colonic distension) between distension 3 and distension 5 (*P* = 0.708, two-way ANOVA with Holm–Šídák’s multiple comparisons test; *N* = 8).

Preincubation with 100 nM IL-13 caused a shift in the afferent pressure-response relationship (*Ramp 5*; Fig. 3*B*), indicating elevated afferent activity during colonic distension (AUC: vehicle, 14.4 ± 1.3 × 10^2^ spikes·s^−1^ mmHg; IL-13, 19.8 ± 2.1 spikes·s^−1^ mmHg; *P* = 0.018, one-way ANOVA with Holm–Šídák’s multiple comparisons test; *N* = 8/group; Fig. 3*C*). IL-13 potentiated afferent firing compared with vehicle treatment at intraluminal pressures ≥60 mmHg (*P* < 0.033, two-way repeated measures ANOVA with Holm–Šídák’s multiple comparisons test; *N* = 8/group; Fig. 3*B*). IL-13 elevated peak afferent firing in response to noxious ramp distension by over 11 spikes·s^−1^ compared with vehicle, with average peak activity of 41.2 ± 7.26 spikes·s^−1^ (*P* = 0.034, one-way ANOVA with Holm–Šídák’s multiple comparisons test; *N* = 8; Fig. 3*D*). Unlike in DRG neuronal cell bodies, where direct stimulation by IL-13 was observed, IL-13 failed to alter basal LSN activity ex vivo (*P* = 0.101, one-way ANOVA with Holm–Šídák’s multiple comparisons test; *N* = 8; Fig. 3*E*), and a similar rate of spontaneous firing was maintained between treatment groups (vehicle: 15.6 ± 5.38 spikes·s^–1^ vs. IL-13: 11.7 ± 3.39 spikes·s^−1^). Therefore, in LSN colonic afferents, IL-13 sensitizes the response to mechanical distension but does not evoke any change in baseline nerve activity.

To assess the requirement for JAK signaling in IL-13-mediated afferent hypersensitivity, the pan-JAK inhibitor pyridone 6 (1 µM) was applied to LSN preparations. JAK inhibition attenuated the effect of IL-13 on distension-evoked afferent activity, blocking the sensitizing effect of IL-13 at intraluminal pressures ≥60 mmHg (*P* < 0.0057, two-way repeated measures ANOVA with Holm–Šídák’s multiple comparisons test; *N* = 7–8; Fig. 3*B*). Afferent firing in tissues treated with both IL-13 and pyridone 6 was no different to vehicle-treated tissues across all intraluminal pressures (*P* > 0.92, two-way repeated measured ANOVA with Holm–Šídák’s multiple comparisons test; *N* = 7–8), and treatment with pyridone 6 alone had no effect compared with vehicle (*P* > 0.26, two-way repeated measures ANOVA with Holm–Šídák’s multiple comparisons test; *N* = 7–8). Both the AUC (*P* = 0.0079, one-way ANOVA with Holm–Šídák’s multiple comparisons test; *N* = 7–8; Fig. 3*C*) and peak afferent firing (*P* = 0.024, one-way ANOVA with Holm–Šídák’s multiple comparisons test; *N* = 7–8; Fig. 3*D*) were suppressed in the presence of pyridone 6 compared with IL-13 alone. Treatment with pyridone 6 had no effect on the baseline spontaneous activity of the LSN when applied with vehicle (*P* = 0.44, one-way ANOVA with Holm–Šídák’s multiple comparisons test; *N* = 7–8; Fig. 3*E*) or IL-13 (*P* = 0.91, one-way ANOVA with Holm–Šídák’s multiple comparisons test; *N* = 7–8; Fig. 3*E*).

Given the dependence of IL-13-evoked Ca^2+^ signals in sensory neurons on p38 MAPK, we also examined the role of this kinase in IL-13-mediated afferent hypersensitivity. Inhibition of p38 MAPK with SB203580 (10 µM) exhibited a similar effect on distension-evoked afferent activity as was observed for JAK inhibition. When applied with SB203580, IL-13 failed to induce a shift in the pressure-response curve (Fig. 3*B*). SB203580 abolished IL-13-evoked mechanical hypersensitivity at distension pressures ≥60 mmHg (*P* < 0.0052, two-way repeated measures ANOVA with Holm–Šídák’s multiple comparisons test; *N* = 6–8; Fig. 3*B*); tissues treated with both IL-13 and SB203580 exhibited afferent firing indistinguishable from the vehicle-treated group at all distending pressures (*P* > 0.28, two-way repeated measures ANOVA with Holm–Šídák’s multiple comparisons test; *N* = 6–8). Furthermore, afferent firing was unchanged at all distending pressures between SB203580- and vehicle-treated groups (*P* > 0.63, two-way repeated measures ANOVA with Holm–Šídák’s multiple comparisons test; *N* = 6–8). The AUC was significantly reduced compared with IL-13 alone (*P* = 0.0020, one-way ANOVA with Holm–Šídák’s multiple comparisons test; *N* = 6–8; Fig. 3*C*). Furthermore, peak afferent firing evoked by IL-13 was also suppressed by p38 MAPK inhibition (*P* = 0.028, one-way ANOVA with Holm–Šídák’s multiple comparisons test; *N* = 6–8; Fig. 3*D*), whereas no effect on baseline firing was observed when SB203580 was applied alone or with IL-13 (*P* = 0.94 and 0.99, respectively, one-way ANOVA with Holm–Šídák’s multiple comparisons test; *N* = 6–8; Fig. 3*E*).

Colonic compliance to ramp distension was measured in each protocol to ensure the same volume of fluid was required to attain an intraluminal pressure of 80 mmHg, thereby ratifying that the treatments did not affect the tone or material properties of the colonic wall. There was no difference in the fluid volume required to reach 80 mmHg intraluminal pressure between ramps 3 and 5 for any of the experimental groups (*P* = 0.78, mixed-effects model with Šídák’s multiple comparisons tests; *N* = 6–8; Fig. 3*F*), nor were there any differences between experimental groups (*P* = 0.21, mixed-effects model with Šídák’s multiple comparisons tests; *N* = 6–8; Fig. 3*F*).

### IL-13 Does Not Sensitize Colonic Afferent Response to Noxious Capsaicin Application

**Figure 4.**
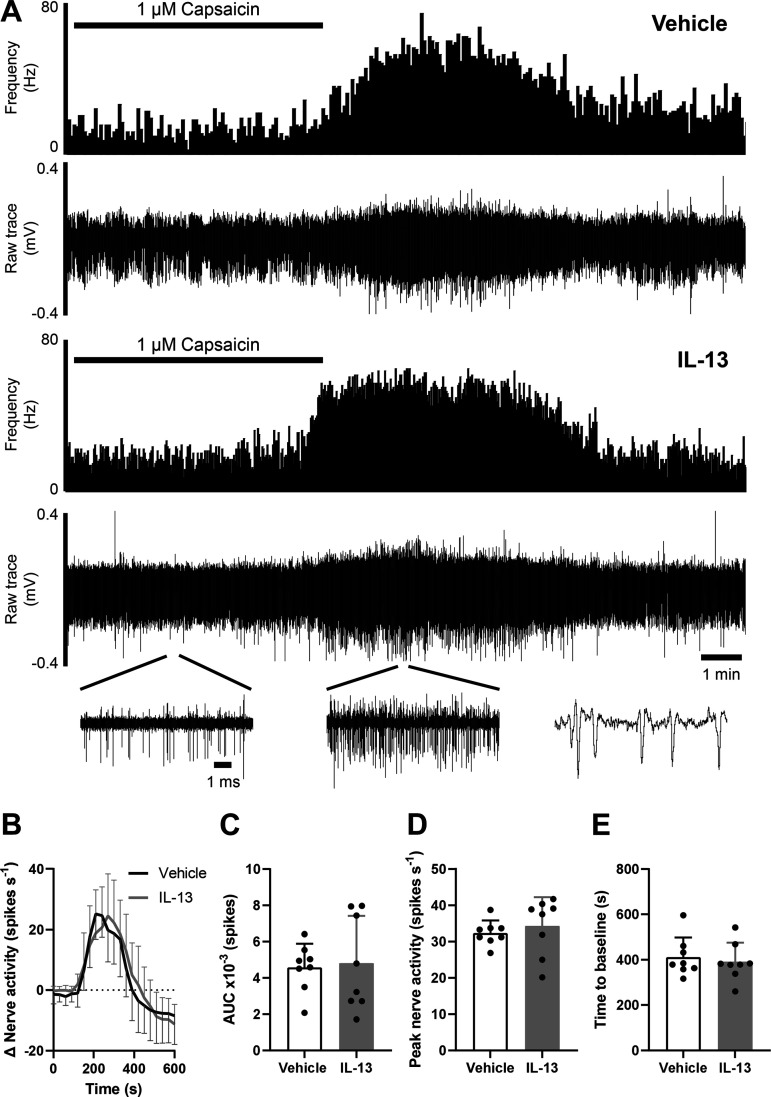
IL-13 does not sensitize the colonic afferent response to capsaicin. *A*: example rate histograms and accompanying neurograms of LSN activity, illustrating similar afferent responses to 1 µM capsaicin after incubation with IL-13 (100 nM) compared with vehicle pretreatment. *B*: afferent firing was unchanged in the 10-min postcapsaicin application (*P* = 0.979, *N* = 8). *C*: IL-13 did not alter response AUC 0- to 10-min postcapsaicin application (*P* = 0.818, unpaired *t* test; *N* = 8). *D*: peak afferent firing remained constant between vehicle- and IL-13-treated tissues (*P* = 0.534, *N* = 8). *E*: the time taken for nerve activity to return to baseline firing following capsaicin application was unchanged by IL-13 incubation (*P* = 0.649, *N* = 8). LSN, lumbar splanchnic nerve.

To determine whether, in addition to sensitizing colonic afferents to mechanical stimuli, IL-13 also sensitized TRPV1-mediated LSN activity, preparations were perfused with 1 µM capsaicin (20 mL). As seen previously, capsaicin produced a direct increase in nerve activity in vehicle-treated tissues (Fig. 4*A*). A similar capsaicin response was observed in preparations treated with 100 nM IL-13, represented by equivalent response AUCs (*P* = 0.818, unpaired *t* test; *N* = 8/group; Fig. 4*B*). IL-13 did not sensitize afferent activity in the 10 min postcapsaicin application (*P* = 0.979, two-way ANOVA with Holm–Šídák’s multiple comparisons test; *N* = 8/group; Fig. 4*C*) and peak afferent firing remained unchanged between treatment groups (vehicle: 32.4 ± 3.44 spikes·s^−1^, IL-13: 34.3 ± 7.92 spikes·s^−1^; *P* = 0.534, unpaired *t* test; *N* = 8/group; Fig. 4*D*). Furthermore, tissues treated with IL-13 (411 ± 87.1 s) and vehicle (392 ± 83.9 s) required the same amount of time to return to basal nerve activity postcapsaicin application (*P* = 0.649, unpaired *t* test; *N* = 8/group; Fig. 4*E*). Together, these data show that, whereas IL-13 sensitized visceral afferents to noxious mechanical distension, IL-13 does not sensitize TRPV1 in colonic afferents.

## DISCUSSION

IL-13 is a proinflammatory cytokine associated with several inflammatory conditions, such as asthma (60), atopic dermatitis (44), and IBD (34). With regard to pain, IL-13 has been observed to both activate and sensitize sensory neurons (44). As seen with TNFα, studies have shown that IL-13-mediated neuronal activation is dependent on extracellular Ca^2+^ entry via TRP channels, including TRPV1 (44). Despite this, no data exists to implicate IL-13 signaling in colonic afferent sensitization, leaving little knowledge of the role of IL-13 in hypersensitivity and visceral pain in GI disease. We therefore investigated and found a pronociceptive role of IL-13 in colonic afferents, which was dependent on p38 MAPK and JAK signaling in the sensitization of responses to noxious mechanical distension. Overall, these data present IL-13 as a potential target for treating pain originating in the colon and highlight the possible benefit of targeting downstream p38 MAPK and JAK signaling pathways to alleviate pain.

First, we observed an IL-13-evoked rise in [Ca^2+^]_i_ in DRG sensory neurons, in line with previous studies (44), the majority of which were small in diameter, thus likely nociceptors. We found that IL-13 activated a greater proportion of DRG neurons compared with previous studies, likely the result of differences in culture conditions, for example, the use of both sexes and inclusion of NGF in DRG neuron cultures can alter chemosensitivity (61). Thus far, the effects of NGF on factors related to IL-13 function in DRG neurons have not been investigated, but if, for example, IL-13Rα2 expression is upregulated, this would result in decreased neuronal stimulation by IL-13 due to its role as a decoy receptor (10). A further indication that IL-13 can stimulate nociceptors arises from the finding that IL-13-sensitive neurons were frequently cosensitive to capsaicin. These findings correlated with scRNA-seq analysis showing high levels of coexpression of TRPV1 and both IL-13 receptor subunits, IL-13Rα1 and IL-4Rα (45). Although Ca^2+^ responses do not necessarily correlate with AP generation, these findings highlight the ability of IL-13 to stimulate sensory neurons and thus the potential for IL-13 modulation in conditions associated with visceral pain.

In TRPV1 knockout mice, IL-13-mediated activation of DRG neurons is reduced, thus implicating TRP channels in IL-13 signaling pathways (44). In support of previous findings, showing that the activation of p38 MAPK and JAK pathways is associated with TRPV1-mediated Ca^2+^ responses in cultured DRG neurons, we confirmed that simultaneous inhibition of JAK1, JAK2, JAK3, and Tyk2 by pyridone 6 reduced the population of IL-13-responsive neurons. More specifically, the effects of IL-13 on DRG neurons were regulated by JAK1/2, since blockade with ruxolitinib attenuated Ca^2+^ responses, recapitulating the effect of pyridone 6. Blockade of p38 MAPK also reduced neuronal stimulation by IL-13, in accordance with previous studies showing activation of p38 MAPK by IL-13 (59). Inhibition of the response to IL-13 by blockade of either p38 MAPK or JAK signaling may indicate an interaction between these pathways. Xu et al. (59) made a similar observation wherein IL-13-induced STAT1/3 phosphorylation was attenuated by p38 MAPK inhibition.

Although the stimulation of DRG sensory neurons in vitro by IL-13 did not translate into direct changes in basal nerve firing ex vivo, IL-13-mediated sensitization of colonic afferents in response to ramp distension was observed for the first time. Increases in afferent firing were noted at pressures ≥60 mmHg, implying that IL-13 sensitized high-threshold mechanonociceptors (i.e., mesenteric and serosal afferents) which correspond to a major class of nociceptor in the GI tract and comprise the majority of LSN afferent subtypes (62). The mechanism through which IL-13 sensitizes colonic afferent neurons is yet unidentified, but somatically, in conditions with elevated levels of IL-13, sensory neuron TRP channel expression, including TRPV1, TRPA1, and TRPV4, is upregulated or the channels themselves are modulated to increase sensitivity (63–65). Furthermore, in cardiomyocytes, IL-13 has been shown to modulate the voltage-gated Na^+^ channels Na_V_1.4 and Na_V_1.5 (66), but no work has thus far investigated if and how IL-13 modulates Na_V_ subunit expression in colonic sensory neurons (e.g., Na_V_1.8; 45). Modulation of these ion channels may also occur in colonic afferent neurons to induce the hypersensitivity seen in response to noxious ramp distension, but further work is needed to understand precisely which channels are involved; for example, investigating whether IL-13 modulates expression or function of putative mechanosensors, such as Piezo2. Piezo2 is frequently coexpressed with IL-13Rα1 and IL-4Rα in lumbosacral DRG neurons innervating the colon (45). It is also important to consider the variety of other cell types present in the gut, which may release pronociceptive mediators in response to stimulation with IL-13. The site of action of IL-13 cannot be fully resolved in our experiments, and so the effects of IL-13 application may arise from both direct and indirect pathways. Defining the role of different cell types in the response to IL-13 is likely to require cell-specific knockout of receptors for IL-13, or the ablation of identified cell types.

Here, we have made the first report of p38 MAPK and JAK signaling in IL-13-induced hypersensitivity of colonic afferent neurons. Previous studies have shown that IL-13 increases p38 MAPK phosphorylation (59) and JAK signaling has been assigned a role in somatic neuronal sensitivity and itch, in which IL-13 plays a key role (44). The distal colon is innervated by a range of sensory afferent populations, each with differing mechanical sensitivities (62). However, ramp distension protocols used in our study only assessed afferent responses to stretch, and although this is most likely the predominant pain-evoking stimulus in GI disease, worsened by neuronal sensitization, further characterization of afferent subtypes sensitized by IL-13 may help to better understand the signaling pathways involved and provide novel targets for intervention. Data presented here show that inhibition of p38 MAPK and JAK signaling had no effect on baseline nerve activity, demonstrating that normal neuronal function is not affected by blocking these pathways and thus may selectively target mechanisms of sensitization to reduce pain in GI disease.

Multiunit recordings of LSN activity showed that IL-13 did not sensitize capsaicin responses and therefore failed to modulate TRPV1 channels in colonic sensory afferents, suggesting other ion channels are responsible for afferent hypersensitivity. This contrasts with TNFα, which was found to sensitize the afferent response to capsaicin (47). IL-13 and TNFα interact with a similar population of TRPV1-expressing colon-innervating neurons and both seem to activate p38 MAPK (45, 47), so why does IL-13 not sensitize TRPV1? SB203580 (used in this study) inhibits both the α and β isoforms of p38 MAPK, which are highly coexpressed in colonic sensory neurons (45). One may speculate that TNFα and IL-13 stimulate different isoforms of p38 MAPK, with TRPV1 being a substrate of only the p38 isoform activated by TNFα. It is also important to consider that the high concentration of capsaicin used in this study (1 µM) may obscure a sensitizing effect of IL-13 by saturating the afferent response or inducing desensitization of TRPV1, though this concentration of capsaicin was used to demonstrate TNFα-induced TRPV1 sensitization (47). TRPA1 has been linked to mechanosensitivity in the gut (67, 68) and, during inflammation, IL-13 increases TRPA1 expression (69). In scRNA-seq analysis, TRPA1 transcripts are present in a subset of TRPV1^+^ neurons, the same population that expresses IL-13Rα1 and IL-4Rα (45). Further functional studies are required to investigate whether the IL-13-mediated colonic afferent hypersensitivity observed in this study is TRPA1-dependent.

In summary, we have shown that IL-13 sensitizes LSN responses to noxious ramp distension, an effect that can be reversed by inhibition of p38 MAPK and JAK signaling pathways. IL-13 receptor expression positively correlates with likely nociceptive populations of colonic afferent neurons (45), making IL-13 and its receptors, alongside downstream p38 MAPK and JAK signaling, feasible targets in the treatment of visceral pain in GI disease.

## DATA AVAILABILITY

All data supporting the results presented in the manuscript are included in the manuscript.

## GRANTS

This work was supported by AstraZeneca PhD Studentship (K.H.B.: RG98186) and the University of Cambridge BBSRC Doctoral Training Program (to J.P.H. and L.A.P.: BB/M011194/1).

## DISCLOSURES

Katie Barker has completed a PhD funded by Astra Zeneca. David Bulmer and Ewan St John Smith have research funding from Astra Zeneca. Fraser Welsh and Iain Chessell are employed by Astra Zeneca. None of the other authors has any conflicts of interest, financial or otherwise, to disclose.

## AUTHOR CONTRIBUTIONS

K.H.B., I.P.C., F.W., E.S.J.S., and D.C.B. conceived and designed research; K.H.B. and J.P.H. performed experiments; K.H.B., J.P.H., L.A.P., and D.C.B. analyzed data; K.H.B., J.P.H., L.A.P., E.S.J.S., and D.C.B. interpreted results of experiments; K.H.B., J.P.H., and D.C.B. prepared figures; K.H.B., J.P.H., E.S.J.S., and D.C.B. drafted manuscript; K.H.B., J.P.H., L.A.P., I.P.C., F.W., E.S.J.S., and D.C.B. edited and revised manuscript; K.H.B., J.P.H., L.A.P., I.P.C., F.W., E.S.J.S., and D.C.B. approved final version of manuscript.
